# Chrysophanic Acid Suppresses Adipogenesis and Induces Thermogenesis by Activating AMP-Activated Protein Kinase Alpha *In vivo* and *In vitro*

**DOI:** 10.3389/fphar.2016.00476

**Published:** 2016-12-08

**Authors:** Hara Lim, Jinbong Park, Hye-Lin Kim, JongWook Kang, Mi-Young Jeong, Dong-Hyun Youn, Yunu Jung, Yong-Il Kim, Hyun-Ju Kim, Kwang Seok Ahn, Su-Jin Kim, Seong-Kyu Choe, Seung-Heon Hong, Jae-Young Um

**Affiliations:** ^1^College of Korean Medicine, Basic Research Laboratory for Comorbidity RegulationKyung Hee University, Seoul, South Korea; ^2^Department of Microbiology and Center for Metabolic Function Regulation, School of Medicine, Wonkwang UniversityIksan, South Korea; ^3^Department of Cosmeceutical Science, Daegu Haany UniversityKyungsan, South Korea; ^4^Department of Pharmacology, College of Pharmacy, Wonkwang UniversityIksan, South Korea

**Keywords:** chrysophanic acid, obesity, adipogenesis, thermogenesis, AMP-activated protein kinase alpha

## Abstract

Chrysophanic acid (CA) is a member of the anthraquinone family abundant in rhubarb, a widely used herb for obesity treatment in Traditional Korean Medicine. Though several studies have indicated numerous features of CA, no study has yet reported the effect of CA on obesity. In this study, we tried to identify the anti-obesity effects of CA. By using 3T3-L1 adipocytes and primary cultured brown adipocytes as *in vitro* models, high-fat diet (HFD)-induced obese mice, and zebrafish as *in vivo* models, we determined the anti-obesity effects of CA. CA reduced weight gain in HFD-induced obese mice. They also decreased lipid accumulation and the expressions of adipogenesis factors including peroxisome proliferator-activated receptor gamma (PPARγ) and CCAAT/enhancer-binding protein alpha (C/EBPα) in 3T3-L1 adipocytes. In addition, uncoupling protein 1 (UCP1) and peroxisome proliferator-activated receptor gamma coactivator 1-alpha (PGC1α), the brown fat specific thermogenic genes, were up-regulated in brown adipocytes by CA treatment. Furthermore, when co-treated with Compound C, the AMP-activated protein kinase (AMPK) inhibitor, the action of CA on AMPKα was nullified in both types of adipocytes, indicating the multi-controlling effect of CA was partially via the AMPKα pathway. Given all together, these results indicate that CA can ameliorate obesity by controlling the adipogenic and thermogenic pathway at the same time. On these bases, we suggest the new potential of CA as an anti-obese pharmacotherapy.

## Introduction

Obesity is a public health dilemma, especially in developed countries which has steadily increased in recent years. The World Health Organization currently estimates that over one billion individuals worldwide are overweight. Almost one-third of these individuals are clinically obese, markedly raising their chances of cardiovascular disease, type 2 diabetes, cancer, and stroke (Waxman, 2004). Even more problematic is that about 25% of children in the USA are also now overweight or obese. These numbers are expected to increase by more than half again by the year 2025 worldwide, with especially severe impact in less developed countries (Haslam and James, 2005).

The regulation of body fat in animals results from the integration of multiple nutrient, sensory, and hormonal inputs primarily at the level of the brain and adipose tissues (Farooqi and O’Rahilly, 2007). The mechanisms underlying the development of obesity may include enzymatic/receptor and hormonal changes (i.e., lipoprotein lipase, hormone sensitive lipase, very low-density lipoprotein (VLDL) receptor, insulin, growth hormone, catecholamine) in the skeletal muscles and adipose tissues. This may result from physical inactivity and inappropriate macronutrient intake (i.e., high levels of saturated fat and/or refined carbohydrates), or both (Garrow, 1998). Thus, the integrated network of obesity is influenced not only by genetics but also by circadian rhythms, as well as physical and social environments (Pospisilik et al., 2010).

Mammals have two types of adipose tissues, white adipose tissues (WAT) and brown adipose tissues (BAT). These two tissues have quite opposite roles in whole-body energy metabolism; that is, WAT is for energy storage, and BAT is for cold and diet-induced thermogenesis, which significantly contributes to the control of body temperature and energy expenditure (Cannon and Nedergaard, 2004). BAT, a site of non-shivering thermogenesis, shows promise in combating obesity, since it contributes to the regulation of whole-body energy expenditure and body fat content in small rodents (Cannon and Nedergaard, 2004). Recent studies using fluorodeoxyglucose-PET in combination with CT revealed that adult humans have considerable amounts of BAT (Cypess et al., 2009; Saito et al., 2009; van Marken Lichtenbelt et al., 2009; Virtanen et al., 2009). BAT thermogenesis is principally dependent on the β-adrenergically mediated activation of lipolysis and subsequent degradation of fatty acids via uncoupling protein 1 (UCP1), which uncouples mitochondrial oxidative phosphorylation to dissipate the electrochemical gradient as heat instead of ATP synthesis. Thus, the β-adrenoceptor–UCP1 system has been expected as an intriguing target for the control of whole-body energy balance, adiposity, and obesity (Lowell and Bachman, 2003; Inokuma et al., 2006; Feldmann et al., 2009).

The 3T3-L1 adipocyte cell line is one of the most well-characterized and reliable models of white adipocytes for studying the conversion of preadipocytes into adipocytes. Adipocytes differentiation is a complex process involving coordinated expression of specific genes and proteins associated with each stage of adipogenesis (Zhou et al., 2009). Differentiation of 3T3-L1 preadipocytes into mature adipocytes is induced by up stimulation with three differentiation inducers (MDI); 3-isobutyl-1-methylxanthine (IBMX), dexamethasone (Dex) and insulin, which promote the accumulation of intracellular lipid droplets in mature adipocytes (Jessen and Stevens, 2002). During adipogenesis of 3T3-L1 cells, peroxisome proliferators activated receptor-γ (PPARγ) and CCAAT/enhancer-binding protein-α (C/EBPα) play key roles as major transcription factors (Lee et al., 2011). The expressions of PPAR, a transcription factor of the nuclear-receptor superfamily and C/EBPα, a member of C/EBP family basic-leucine zipper class of transcription factors are increased during differentiation of 3T3-L1 cells (Kwak et al., 2012). Lipin is also a central regulator of adipose tissue development. Mammalian lipin proteins have been shown to enzymatically convert phosphatidate to diacylglycerol, an essential precursor in triacylglycerol and phospholipid synthesis (Ugrankar et al., 2011). A study by Zhang et al. (2012) established that the *Lipin1* gene is required at an early step in adipocyte differentiation for induction of the adipogenic gene transcription program, including the key regualtor PPARγ.

AMP-activated protein kinase (AMPK), composed of α, β, and γ subunits, is a “cellular fuel gage” which acts to simultaneously shut down ATP-consuming biosynthetic processes and facilitate ATP-producing catabolic processes during periods of metabolic stress, leading to rapid changes in the control of fatty acid metabolism. Thus, AMPK is a key player in energy homeostasis. AMPK-derived stimulation of fatty acid metabolism occurs as a result of AMPK phosphorylation (Kim and Choung, 2012). AMPK is phosphorylated when the intracellular AMP/ATP ratio increases because of metabolic stress. Subsequently, downstream target molecules are activated, resulting in promotion of catabolism. On the other hand, when the intracellular AMP/ATP ratio decreases, AMPK increases anabolism. This key regulator of energy homeostasis is also associated with adipocyte differentiation in 3T3-L1 cells (Kim and Kong, 2010; Huang et al., 2011).

Chrysophanic acid (CA) is a member of the anthraquinone family. The results of previous pharmaceutical studies have shown that derivatives of anthraquinones exert a number of biological effects, including anticancer (Shoemaker et al., 2005; Huang et al., 2007), hepatoprotective (Arosio et al., 2000), antimicrobial (Fosse et al., 2004), and anti-inflammatory (Kim et al., 2010). As several studies have indicated that obesity is associated with a low-grade proinflammatory state (Weisberg et al., 2003; Hajer et al., 2008), the anti-inflammatory feature of CA suggests the possible use of CA in obesity-related issues. In addition, several studies also report the use of rhubarb in obesity care (Greenway et al., 2006; Amin and Nagy, 2009; Tseng et al., 2011; Zhou et al., 2014), a herb which CA is one of the active compounds (Liu et al., 2005). However, even though numerous biological activities of CA have been reported, there is only limited evidence for its anti-obesity effect. In this study, we investigated how CA works on adipocyte differentiation process at the molecular levels in 3T3-L1 adipocytes and primary cultured brown adipocytes, and the *in vivo* effects of dietary CA on body weight changes, physiological, histological, and metabolic variables in high-fat diet (HFD)-fed C57BL/6J mice were evaluated.

## Materials and Methods

### Chemical Reagents

Chrysophanic acid (>98% pure) was purchased from Sigma Chemicals (St. Louis, MO, USA) and was dissolved in 100% dimethyl sulfoxide (DMSO, Sigma Chemicals, St. Louis, MO, USA). The Dulbecco’s modified Eagle’s medium (DMEM) and penicillin-streptomycin were from Gibco BRL (Grand Island, NY, USA). Fetal bovine serum (FBS) was from HyClone (Logan, UT, USA). Antibodies for PPAR-γ, AMPKα, and p-AMPKα were purchased from Cell Signaling Technology (Beverly, MA, USA), antibodies for C/EBP-α, Glyceraldehyde-3-phosphate dehydrogenase (GAPDH), and PPAR-γ coactivator 1-alpha (PGC1α) were purchased from Santa Cruz Biotechnology (Santa Cruz, CA, USA), and antibody for UCP1 was from Millipore Corporation (Bedford, MA, USA).

### Animals and Diets

Male 4-week-old C57BL/6J mice were purchased from Daehan Biolink Co. (Eumsung, Korea) and maintained for 1 week prior to experiments. Mice were maintained on a 12-h light/dark cycle in a pathogen-free animal facility, provided with laboratory diet and water *ad libitum*. All experimental protocols involving the use of animals were conducted under approval of the Animal Care and Use Committee of the Institutional Review Board of Kyung Hee University (confirmation number : KHUASP(SE)-12-036). To induce obesity, the mice were fed a HFD (Rodent diet D12492; Research diet, New Brunswick, NJ, USA) with 60% kcal% fat. Control group (C) were fed a commercial standard chow diet (CJ Feed Co., Seoul, Korea). HFD group (HFD) mice were fed with HFD only. HFD plus CA group (CA) Mice were fed with HFD for 4 weeks before administration of CA (5 mg/kg/day). The mice were divided into three groups (*n* = 5) that were fed chow diet, HFD, and HFD plus CA for 16 weeks. Body weight and food intake were measured three times per week.

### Serum Analysis

Plasma was separated immediately after blood sampling by centrifugation at 4,000 *g* for 30 min. Total cholesterol (TC), high-density lipoprotein (HDL) cholesterol, LDL cholesterol, triglyceride, glucose, aspartate aminotransferase (AST), and alanine aminotransferase (ALT) were assessed using enzymatic colorimetric methods performed by Seoul Medical Science Institute (Seoul Clinical Laboratories, Seoul, Korea).

### Hematoxylin and Eosin (H&E) Staining

After the mice were sacrificed, the white fat tissues, brown fat tissues and liver tissues were collected, washed in saline and fixed in 10% formalin. The formalin-fixed, paraffin-embedded prostate specimens were cut into 4-μm-thick tissue sections. These sections were deparaffinized in xylene and rehydrated in serial alcohol. After treatment of 150 μl 0.1% trypsin working solution (consisted of trypsin 0.4 ml, calcium 0.01 g, chloride 0.01 g in D.W. 7 ml) for 15 min, the sections were blocked using FBS. For H&E staining, the sections were stained in hematoxylin for 5 min, and then washed with water for 5 min. Then the sections were stained in eosin for 30 s, dehydrated, and mounted by routine methods.

### Mitochondrial Microscopic Analysis

To label the mitochondria, the mature brown adipocytes were incubated with Mito-Tracker Red probes CM-XRos (Invitrogen, Carlsbad, CA, USA). The cells were fixed and permeabilized after incubation in staining solution containing Mito-Tracker probe for 30 min. Fluorescence signals were imaged using an IX71 confocal microscope (Zeiss, Germany).

### Zebrafish Maintenance and Treatment

Wild type zebrafish was handled and maintained according to the standard protocol (Kimmel et al., 1995). Larvae obtained from daily crosses were raised and fed regular diet starting at 6 days post fertilization (dpf). Treatment of either DMSO or CA (at 10 μM final concentration) was done for 8 days starting from 10 to 17 dpf (*n* = 15 per group). To visualize adipocytes, larvae were treated with Nile red (Sigma Chemicals, St. Louis, MO, USA) and imaged with a Leica M165FC microscope (Leica Microsystems, Wetzlar, Germany). The intensity of fluorescent signals was quantified by ImageJ 1.47v (National Institute of Health, Bethesda, MD, USA).

### Cell Culture and Adipocyte Differentiation

Cell culture, differentiation of 3T3-L1 preadipocytes (American Type Culture Collection, Manassas, VA, USA) were performed as previously reported (Jeong et al., 2015). Briefly, 3T3-L1 pre-adipocytes were cultured in six-well plates until confluence in DMEM containing 1% penicillin–streptomycin and 10% BS at 37°C in 5% CO_2_ atmosphere. Two days after full confluence (day 0), cells were stimulated to differentiate for 2 days with differentiation inducers [0.5 mM IBMX, 1 μM Dex, 1 μg/ml insulin (MDI)] which were supplemented to DMEM containing 25 mM glucose and 10% FBS. From day 2 to 4, the cells were incubated with DMEM containing 10% FBS, 1 μg/ml insulin and various concentrations of CA added to the medium. From day 4 to 6, the medium (consisting of DMEM with 10% FBS and 1 μg/ml insulin) was changed.

Brown adipocytes were obtained from the interscapular BAT of mice (post-natal day 2) and isolated, cultured and differentiated as previously described (Jeong et al., 2015). Briefly, cells were grown in DMEM containing 20% FBS and 1 M HEPES at 37°C in a 5% CO_2_ atmosphere. Two days after full confluence (day 0), were differentiated by incubation for 2 days in differentiation media (DM), which is DMEM containing 25 mM glucose, 0.5 mM IBMX, 0.5 μM Dex, 20 nM insulin, 125 μM indomethacin, 1 nM T3, and 10% FBS. After 48 h (day 2), the cells were incubated with DMEM containing 10% FBS, 20 nM insulin, 1 nM T3, and various concentrations of CA added to the medium. From day 4 to 6, the medium (consisting of DMEM with 10% FBS, 20 nM insulin, and 1 nM T3) was changed every 2nd day.

### MTS Cell Viability Assay

3T3-L1 preadipocytes and primary cultured brown adipocytes were seeded in 96 well plates (2 × 10^4^ cells/well) and incubated for 24 h. Then the cells were incubated in 10% FBS/DMEM medium containing CA for additional 48 h. Cell viability was monitored by the Cell Proliferation MTS Kit (Promega Co., Madison, WI, USA) as recommended by the manufacturer. The absorbance was measured at 490 nm in a VERSAmax microplate reader (Molecular Devices, Sunnyvale, CA, USA) to determine the formazan concentration, which is proportional to the number of live cells.

### Oil Red O Staining

Intracellular lipid accumulation was measured using the Oil Red O method as described by Ramirez-Zacarias et al. (1992). To quantify the intracellular lipids, the stained lipid droplets were dissolved in isopropanol and read for absorbance at 500 nm with a VERSAmax microplate reader (Molecular Devices, Sunnyvale, CA, USA).

### RNA Extraction and Real-Time RT-PCR

Real-time RT-PCR analyses were performed described previously (Jeong et al., 2015). Briefly, total RNA from tissues and cells were isolated using a QIAzol lysis reagent (QIAGEN sciences Inc., Venlo, Netherlands) and a GeneAll^R^ RiboEx Total RNA extraction kit (GeneAll Biotechnology, Seoul, Korea). Total RNA was used as a template for first strand cDNA synthesis with a Power cDNA synthesis kit (iNtRON Biotechnology, Seoul, Korea), and Step One Plus Real-Time PCR System (Applied Biosystems, Foster City, CA, USA) was used for PCR analyses. The primers used in the experiments are shown in **Table 1**.

**Table 1:** The primer sequences used for real-time RT-PCR.

Target gene	Primer sequences
*Pparg*	5′-TTTCAAGGGTGCCAGTTTC-3′ (sense)
	5′-TTATTCATCAGGGAGGCCAG-3′ (antisense)
*Cebpa*	5′-GCCGAGATAAAGCCAAACAA-3′ (sense)
	5′-CGTAAATGGGGATTTGGTCA-3′ (antisense)
*Glut4*	5′-CGAGCTGGACGACGGACACTC-3′ (sense)
	5′-AGACATAGCTCATGGCTGGAACCCG-3′ (antisense)
*Lipin1*	5′-TTCCTTGTCCCTGAACTGCT-3′ (sense)
	5′-TGAAGACTCGCTGTGAATGG-3′ (antisense)
*aP2*	5′-CGTAAATGGGGATTTGGTCA-3′ (sense)
	5′-TCGACTTTCCATCCCACTTC-3′ (antisense)
*Sirt1*	5′-AGTTCCAGCCGTCTCTGTGT-3′ (sense)
	5′-GATCCTTTGGATTCCTGCAA-3′ (antisense)
*Ucp1*	5′-AACTGTACAGCGGTCTGCCT-3′ (sense)
	5′-TAAGCCGGCTGAGATCTTGT-3′ (antisense)
*Sirt3*	5′-TCGAAGGAA AGATGTGGTCC-3′ (sense)
	5′-ATCTGTCCTGTCCATCCAGC-3′ (antisense)
*Pgc1a*	5′-AATGCAGCGGTCTTAGCACT-3′ (sense)
	5′-TGTTGACAAATGCTCTTCGC -3′ (antisense)
*Prdm16*	5′-TGGGCTCACTACCCTACCAC-3′ (sense)
	5′-GACTTTGGCTCAGCCTTGAC-3′ (antisense)
*Gapdh*	5′-AACTTTGGCATTGTGGAAGG-3′ (sense)
	5′-GGATGCAGGGATGATGTTCT-3′ (antisense)


### Western Blot Analysis

Western blot analyses were performed described previously (Jeong et al., 2015). Harvested cells were lysed with ice-cold RIPA buffer and then centrifuged at 13,000 rpm for 20 min at 4°C to remove the insoluble materials. Next, the total concentration of extracted proteins was determined using the method based on the study of Bradford (1976). The proteins in the supernatants were separated by 8% sodium dodecyl sulfate-polyacrylamide gel electrophoresis and transferred onto Polyvinylidene difluoride (PVDF) membranes. After blocking with 10 mM Tris, 150 mM NaCl, and 0.05% Tween-20 (TBST) containing 5% skim milk for 1 h at room temperature, the membranes were washed with TBST and then incubated with the primary antibody at 4°C overnight. The blots were subsequently incubated with horseradish peroxidase (HRP)-conjugated affinipure Goat anti-rabbit IgG (Jackson Immunoresearch lab., West Grove, PA, USA) or HRP-conjugated affinipure Goat anti-mouse IgG (Jackson Immunoresearch lab., West Grove, PA, USA). PVDF membranes were purchased from Millipore (Bedford, MA, USA), and the protein assay reagent was obtained from Bio-Rad (Hercules, CA, USA).

### Statistical Analysis

All data, expressed as mean ± standard deviation (SD), were processed statistically by IBM SPSS Statistics 22 software (International Business Machines Corp., New York, NY, USA). Values with *p* < 0.05 were considered to indicate statistical significance.

## Results

### CA Improves HFD-Induced Obesity in C57BL/6 Mice

**FIGURE 1:**
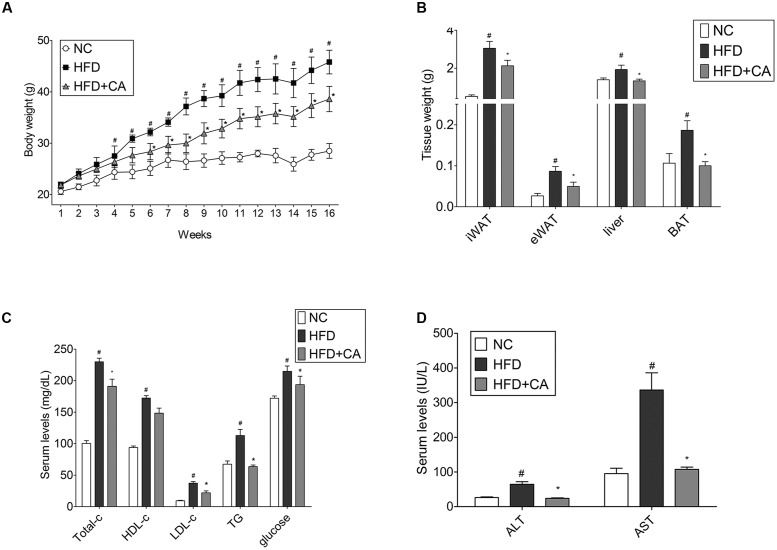
**Chrysophanic acid suppresses weight gain in HFD-induced obese C57BL/6J mice.** **(A)** Weight changes for 16 weeks, **(B)** iWAT, eWAT, liver, and BAT tissue weights were measured. **(C)** Total cholesterol, HDL-cholesterol, LDL-cholesterol, triglyceride, glucose, **(D)** AST, and AST of blood serum were measured. Data represent means ± SD. *^#^p* < 0.05 compared with NC, *^∗^p* < 0.05 compared with HFD. NC, normal control group; HFD, high-fat diet group; HFD + CA, high-fat diet plus CA group (*n* = 5 per group).

**FIGURE 2:**
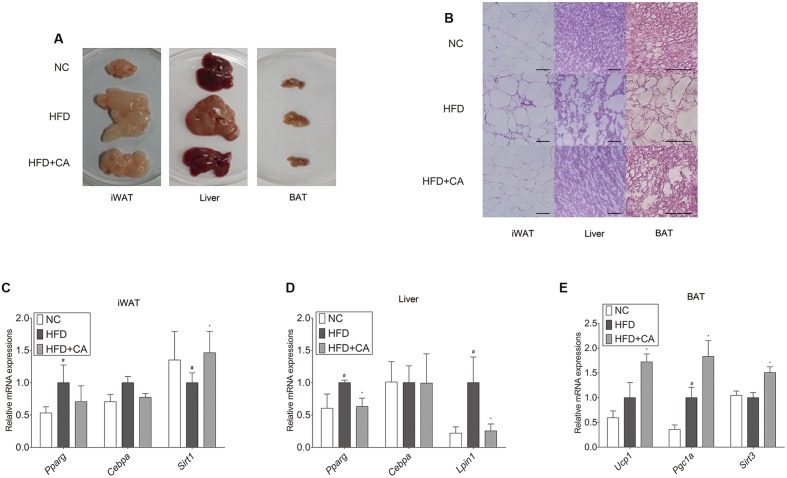
**Chrysophanic acid shows inhibitory effects on adipogenesis-related factors in tissues of C57BL/6J mice.** **(A)** Visual comparisons and **(B)** H&E stainings of iWAT (magnification 200×, scale bar 100 μm), liver (magnification 200×, scale bar 100 μm), and BAT (magnification 400×, scale bar 100 μm) were performed. Real-time RT-PCR analyses of **(C)** *Pparg*, *Cebpa*, and *Sirt1* in iWAT, **(D)** *Pparg*, *Cebpa*, and *Lipin1* in liver, and **(E)** *Ucp1*, *Pgc1a*, and *Sirt3* in BAT were performed. GAPDH was used as endogenous control. Data represent means ± SD of three independent experiments. *^#^p* < 0.05 compared with NC, *^∗^p* < 0.05 compared with HFD. NC, normal control group; HFD, high-fat diet group; HFD + CA, high-fat diet plus CA group (*n* = 5 per group).

The *in vivo* performance of CA was performed in male C57BL/6J mice to determine the efficacy of administered CA. Experimental animals appeared healthy, showing no pathological signs or abnormalities during the feeding period. As shown in **Figure 1A**, the three groups had similar body weights at the beginning of the study. However, mice fed the HFD gained significantly more weight than those fed the standard diet mice. On the other hand, weight gain of CA group was significantly less than with the untreated HFD. Mice in the HFD-group gained 23.92 ± 1.74 g of weight, while those in the CA group gained 16.72 ± 2 g of weight after 16 weeks. There was no difference between HFD and HFD + CA in food intake (data not shown). **Figures 1B** and **2A** show the efficacy of CA on both mass and size of inguinal WAT (iWAT), epididymal WAT (eWAT), liver, and BAT tissues.

After the 16-week experiment, plasma parameter analysis was conducted. As shown, the level of TC, LDL-cholesterol, triglyceride, glucose, AST, and ALT were changed significantly (**Figures 1C,D**). However, there were no differences in HDL-cholesterol levels. Furthermore, the most interesting thing was that during the long-term of treatment with CA, ALT, and AST, which are factors known to indicate hepatotoxicity (Yang et al., 1997), were also brought within normal range. This indicates that CA can ameliorate the hepatic toxicity caused by obesity.

The histological study of iWAT, liver, and BAT (**Figure 2B**) showed fat accumulation in HFD-fed group. In contrast, HFD+CA group did not show increased fat accumulation, even though they were also fed HFD. Moreover, *Pparg*, *Cebpa*, *Lipin1*, *Ucp1*, and *Sirt3* (Schwer et al., 2002) were evaluated in tissues (**Figures 2C–E**). As in **Figure 2C**, there were no significant differences by CA treatment from HFD group in iWAT, except for the increase of *Sirt1*. However, *Pparg* and *Lipin1* were suppressed significantly in the liver (**Figure 2D**) while *Ucp1*, *Pgc1a*, and *Sirt3* were highly elevated in BAT (**Figure 2E**).

### CA Suppresses Lipid Accumulation in Zebrafish

**FIGURE 3:**
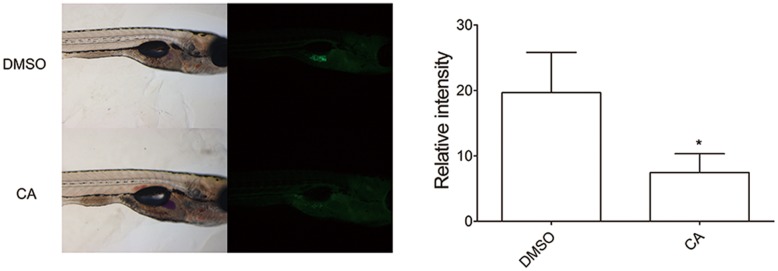
**Chrysophanic acid impairs adipocyte development in zebrafish.** Zebrafish larvae treated with either DMSO (10 μM) or CA (10 μM) were shown in lateral views with anterior to the right. Both bright-field **(Left)** and fluorescent images **(Right)** were paired to visualize the location of adipocytes detected by Nile red. The quantification of the signal intensity from Nile red-positive adipocytes was measured by making multi-point selections of adipocytes using ImageJ. *^∗^p* < 0.05 compared with DMSO-treated control. DMSO, DMSO-treated control group; CA, CA-treated group (*n* = 15 per group).

To further verify the *in vivo* effect of CA in adipocyte development, we next examined developing zebrafish larvae after CA treatment (**Figure 3**). We used the zebrafish as another *in vivo* model to test the effect of CA during adipose tissue development. Recently, development of adipose tissue in zebrafish has recently been well characterized and also been shown to depend highly on nutritional status (Flynn et al., 2009; Imrie and Sadler, 2010; Minchin and Rawls, 2011). Based on the published studies, we verified the effect of CA on zebrafish adipogenesis, which we think highlights a robust effect of CA on adipogenesis across species. Adipose tissues in normally developing zebrafish initially appear in the pancreatic depot at 12 dpf followed by in the visera at 17 dpf, subcutaneous regions at 20 dpf, and the cranium at 22 dpf (Imrie and Sadler, 2010). CA (10 μM) or DMSO as a control was treated from 10 to 17 dpf and adipocytes of CA-treated group compared to those of DMSO-treated control group were examined. As shown in **Figure 3**, CA treatment significantly reduced the degree of fluorescent intensity, suggesting that CA may impair adipocyte development by suppressing accumulation of neutral lipids in developing zebrafish larvae, consistent with our previous *in vivo* results of mice.

### CA Inhibits Lipid Accumulation in 3T3-L1 Adipocytes

**FIGURE 4:**
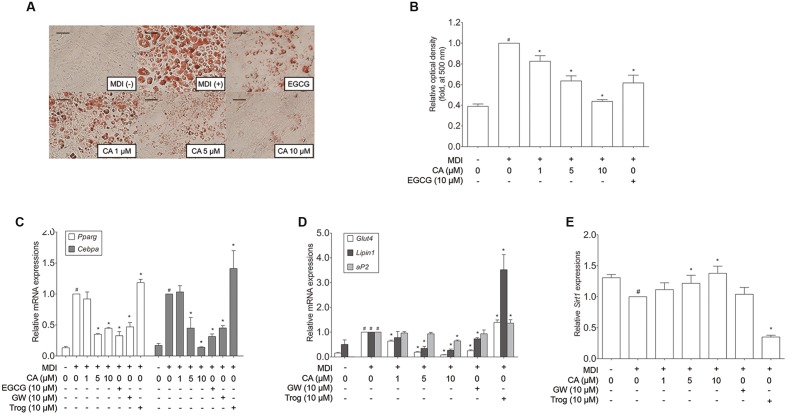
**Chrysophanic acid suppresses adipogenic factors in 3T3-L1 adipocytes.** **(A)** An Oil Red O analysis was performed in order to measure the lipid accumulation (magnification 200×, scale bar 100 μm). **(B)** Relative optic density was measured at 500 nm. Real-time RT-PCR analyses of **(C)** *Pparg*, *Cebpa*, **(D)** *Glut4*, *Lipin1*, *aP2*, and **(E)** *Sirt1* were performed. EGCG (10 μM) and GW (10 μM) were used as positive controls. Trog (10 μM) was used as a negative control. GAPDH was used as endogenous control. Data represent means ± SD of three independent experiments. ^#^*p* < 0.05 compared with MDI-uninduced preadipocytes, *^∗^p* < 0.05 compared with MDI-induced adipocytes. MDI, differentiation medium.

In order to assess the cell autonomous effects of CA, further investigations concerning *in vitro* models were carried out, using 3T3-L1 adipocytes and primary cultured brown adipocytes. To determine the cytotoxicity of CA, 3T3-L1 preadipocytes were treated with various concentrations (1–10 μM) of CA, after which cell viability was measured by using the MTS assay. When measured, treatment with 1–10 μM of CA did not cause significant cytotoxic effects for 3T3-L1 cells (data not shown). Due to this result, further investigations were proceeded at the specific concentrations of 1, 5, and 10 μM. Next, to investigate the effects of CA on adipogenesis, the lipid accumulation was measured by an Oil Red O staining assay. As shown in **Figures 4A,B**, treatments with 1, 5, and 10 μM of CA suppressed lipid accumulation in 3T3-L1 adipocytes with statistical significances (*p* < 0.05), suggesting that CA inhibits adipogenesis. Epigallocatechin gallate (EGCG) was used as a positive control.

### CA Down-Regulates Adipogenic Factors in 3T3-L1 Adipocytes

**FIGURE 5:**
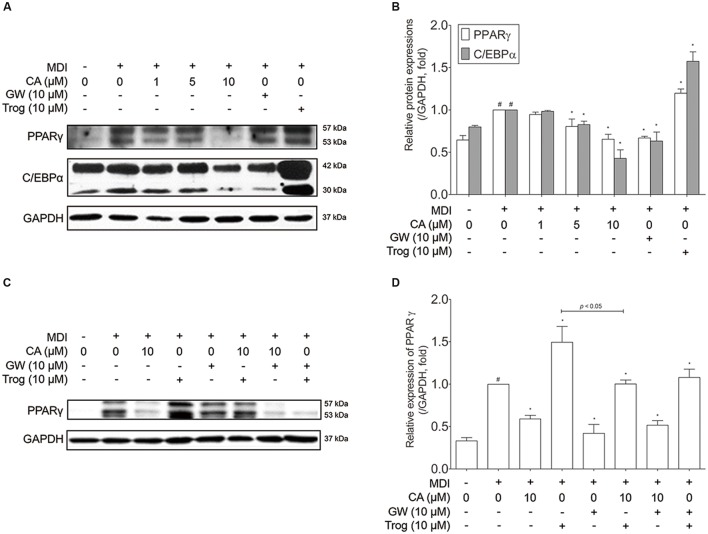
**Chrysophanic acid suppresses PPARγ in competition against the PPARγ agonist troglitazone in 3T3-L1 adipocytes.** **(A)** Western blot analyses of PPARγ and C/EBPα were performed and **(B)** quantification of the protein bands was measured using ImageJ. **(C)** Western blot analysis of PPARγ was performed under co-treatment of CA with GW or Trog, and **(D)** quantification of the protein bands was measured using ImageJ. EGCG (10 μM) and GW (10 μM) were used as positive controls. Trog (10 μM) was used as a negative control. GAPDH was used as endogenous control. Data represent means ± SD of three independent experiments. ^#^*p* < 0.05 compared with MDI-uninduced preadipocytes, *^∗^p* < 0.05 compared with MDI-induced adipocytes. MDI, differentiation medium.

Adipocyte differentiation accompanies the changes in expression of various adipogenic and lipogenic genes such as *Pparg*, *Cebpa*, *Glut4* (Park et al., 2011), *Lipin1*, *aP2* (Gregoire et al., 1998), and *Sirt1* (Picard et al., 2004). In order to evaluate the effect of CA on gene expression in 3T3-L1 adipocytes, fully differentiated 3T3-L1 cells were exposed for 48 h to various concentrations (1, 5, and 10 μM) of CA, and the results were compared to positive control groups, treated with EGCG or GW9662 (GW), and a negative control group treated with troglitazone (Trog). Expressions of adipogenic genes *Pparg*, *Cebpa*, *Glut4*, *Lipin1*, and *aP2* were significantly decreased by CA (**Figures 4C,D**). In addition, CA significantly increased the expression of *Sirt1* in a dose-dependent manner (**Figure 4E**). CA treatment also suppressed PPARγ and C/EBPα at the protein levels (**Figures 5A,B**). To further demonstrate the effects of CA, we co-treated CA with PPARγ agonists or antagonists during the adipogenesis process of 3T3-L1 adipocytes (**Figures 5C,D**). PPARγ was decreased by CA; whether as a sole treatment, or used together with the PPARγ antagonist, GW. In addition, CA significantly nullified the effect of Trog which implies that CA can compete against the activated adipogenesis by Trog, the PPARγ agonist.

### CA Induces Thermogenic Factors in Primary Cultured Brown Adipocytes

**FIGURE 6:**
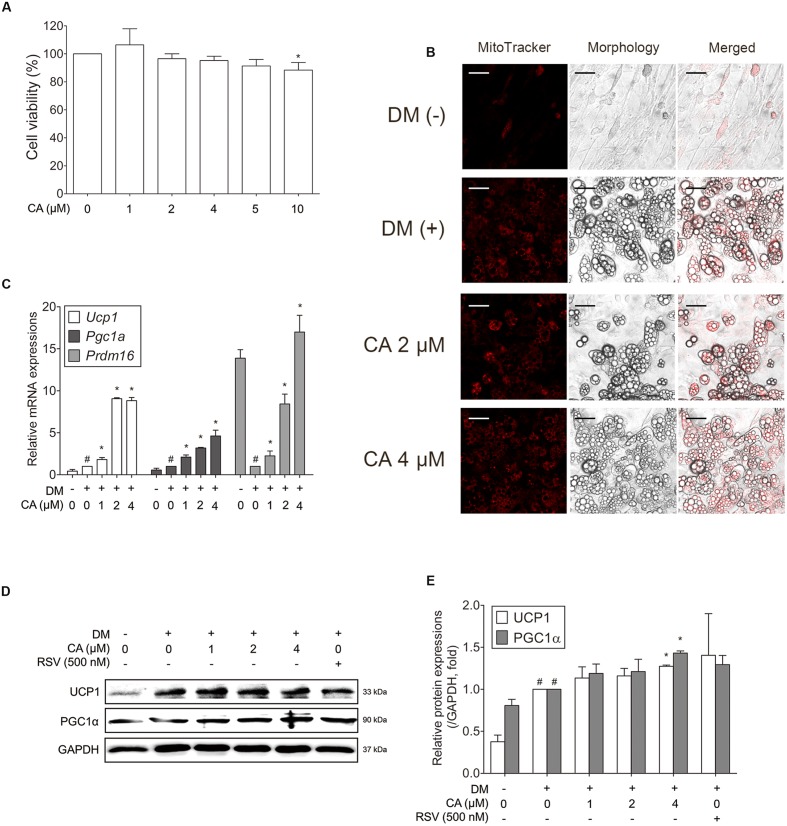
**Chrysophanic acid upregulates brown-fat-specific factors at both mRNA and protein levels in primary cultured brown adipocytes.** **(A)** An MTS assay was performed to measure the survival rate of primary cultured brown adipocytes after treatment with CA. **(B)** Mitochondrial abundance in primary brown adipocytes was analyzed by MitoTracker Red staining (magnification 200×, scale bar 100 μm). **(C)** Real-time RT-PCR analyses of *Ucp1*, *Pgc1a*, and *Prdm16* were performed. **(D)** Western blot analyses of UCP1 and PGC1α were performed. **(E)** Quantification of the protein bands was measured using ImageJ. RSV (500 nM) was used as a positive control. GAPDH was used as endogenous control. Data represent means ± SD of three independent experiments. *^#^p* < 0.05 compared with DM-uninduced preadipocytes, *^∗^p* < 0.05 compared with DM-induced adipocytes. DM, differentiation medium.

Through the mice experiment results, we could infer the probable interaction between CA and BAT. To certify the interaction between CA and brown adipocytes, we conducted experiments on primary cultured brown adipocytes. To determine the cytotoxicity of CA in brown adipocytes, they were treated with CA at various concentrations (1–10 μM) and the cell viability was measured by the MTS assay. Due to the result, further investigations were proceeded at the specific concentrations of 1, 2, and 4 μM (**Figure 6A**).

Since the activity and number of mitochondria is a typical difference between white and brown adipocytes, the mitochondrial abundance was also analyzed using Mito-Tracker fluorescence images. As shown in **Figure 6B**, CA-treated cells showed stronger staining than in control cells in a concentration-dependent manner.

To investigate the efficacy of CA in thermogenesis activation of brown adipocytes, thermogenic genes *Ucp1*, *Pgc1a* (Puigserver et al., 1998), and *Prdm16*, the transcription factor of brown fat (Seale et al., 2007), were evaluated. **Figure 6C** shows significant effects of CA on gene expressions in brown adipocytes. Additionally, UCP1 and PGC1α were increased significantly at the protein levels as well (**Figures 6D,E**). Resveratrol (RSV), a nature-derived compound reported to activate BAT thermogenesis (Andrade et al., 2014) was used as positive control.

### CA Suppresses Adipogenesis and Induces Thermogenesis via Activation of AMPK Pathway

**FIGURE 7:**
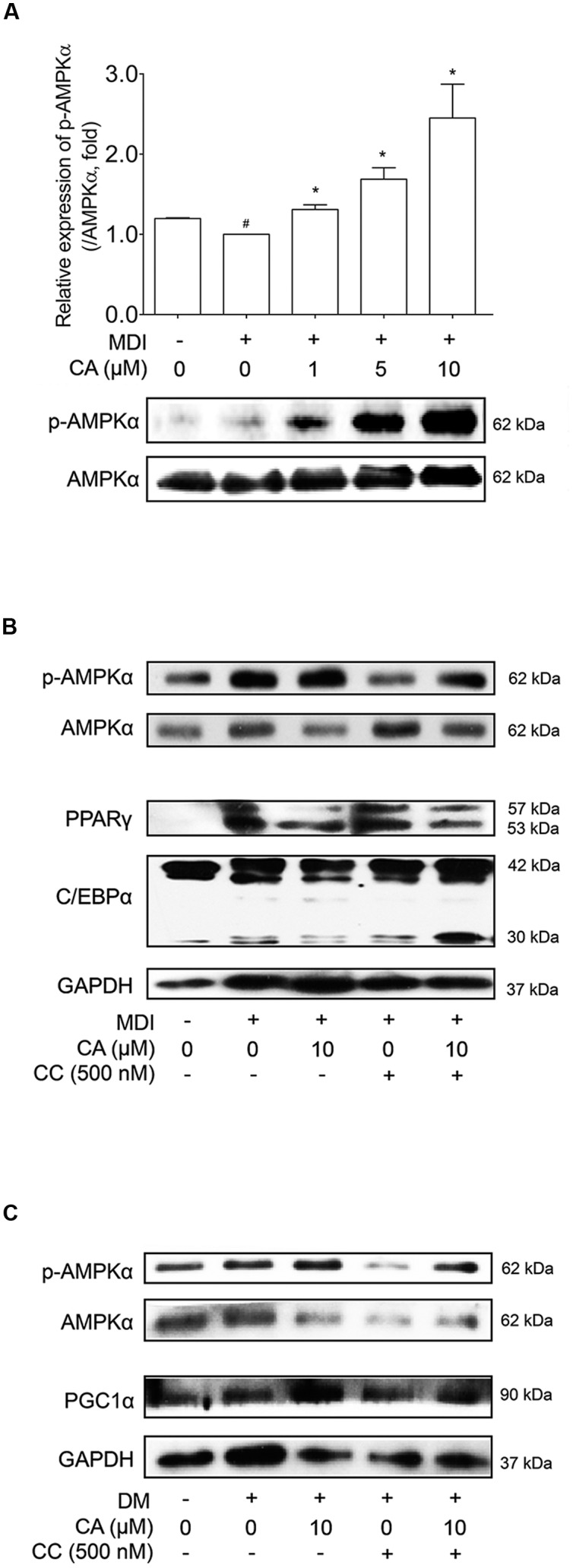
**Chrysophanic acid induces AMPK phosphorylation resulting in inhibition of adipogenesis in 3T3-L1 adipocytes and activation of thermogenesis in primary cultured brown adipocytes.** **(A)** Western blot analyses of p-AMPKα and AMPKα were performed in CA-treated 3T3-L1 adipocytes. **(B)** Western blot analyses of p-AMPKα and AMPKα were performed under co-treatment of CA and CC in 3T3-L1 adipocytes. **(C)** Western blot analyses of p-AMPKα and AMPKα were performed under co-treatment of CA and CC in primary cultured brown adipocytes. The phosphorylation level of AMPKα was measured by p-AMPKα/AMPKα rate. GAPDH was used as endogenous control. Data represent means ± SD of three independent experiments. *^#^p* < 0.05 compared with MDI- or DM-uninduced preadipocytes, *^∗^p* < 0.05 compared with MDI- or DM-induced adipocytes. MDI and DM, differentiation medium.

As CA showed elevated SIRT1 and SIRT3 levels *in vivo* and *in vitro*, its possibility as an AMPK activator led us to additional investigation concerning the effects of CA on the AMPK pathway. The relationship between SIRT1 and AMPK is already well reported (Ruderman et al., 2010), and SIRT3, another protein belonging to the sirtuin family, also activates AMPK and PGC1α as well (Palacios et al., 2009). Additionally AMPK, a key player in energy homeostasis (Lowell, 1999), was activated by CA during adipocyte differentiation, when the level of phosphorylated AMPKα was analyzed and compared with the total level of AMPKα. When compared with the control group, AMPKα phosphorylation was increased in a dose-dependent manner of CA treatment (**Figure 7A**), while the total AMPKα was unchanged.

In **Figure 7B**, Compound C (CC), the AMPK inhibitor, suppressed activation of AMPK, resulting in increased protein levels of PPARγ and C/EBPα. Under inhibited AMPK conditions by CC treatment, the suppressing action of CA on these proteins was nullified. Additional experiments concerning CC were performed in primary cultured brown adipocytes (**Figure 7C**). Similar to the results from 3T3-L1 cells, by blocking AMPK activation, CA failed to increase the expression of PGC1α. Through these results, we could confirm the anti-obese effect of CA by inhibiting adipogenesis in WAT and inducing thermogenesis in BAT was partially via activation of the AMPK pathway.

## Discussion

Anti-obesity drugs currently available are plagued with inefficacy and side-effects ranging from mild to potentially harmful (Gooda Sahib et al., 2012; Rodgers et al., 2012). Consequently, the use of natural products to combat obesity is rapidly increasing. Research to identify targets from natural and alternative sources for development into clinical treatments against obesity is gaining momentum (Gooda Sahib et al., 2012). CA, a yellow crystalline substance extracted from rhubarb, which is frequently used in Korean Medicine for various purposes including treatment for obesity. Despite the facts previous studies have shown numerous effects of CA, not one study involving anti-obesity effects of CA has been reported to date.

Obesity is defined as increased adipose accumulation of excess energy resulting from imbalance between intake and expenditure of energy (Stunkard, 1996). C57BL/6 mice fed with HFD is a widely used experimental model for obesity research. Although genetically engineered mice models such as *ob/ob* (mice unable to produce leptin) or *db/db* (mice unable to respond to leptin) are available for obesity research, they lack convincing evidence for whether leptin-related mutation can represent typical obesity in humans (Wang et al., 2014). As shown by Lin et al. (2000), long-term HFD administration in C57BL/6 mice results in obesity development, showing increased body weight gain and fat accumulation. We conducted an *in vivo* model referring this report, in order to evaluate the anti-obese effect of CA. As a result, CA treatment successfully inhibited weight gain, lipid accumulation in WATs, liver, and BAT. It also ameliorated obese- and hepatotoxic-related changes in serum caused by HFD administration. Assays regarding mRNA expressions in tissues showed decreased expressions of adipogenic genes such as *Pparg*, *Cebpa*, *Sirt1*, and *Lpin1*, while thermogenic genes including *Ucp1* and *Pgc1a* were increased.

PPARγ and C/EBPα are key transcription factors in adipogenesis of 3T3-L1 cells (Lee et al., 2011). C/EBPβ is induced immediately after differentiation, whereas C/EBPα and PPARγ are expressed much later (Cristancho and Lazar, 2011). They are necessary for the expression of adipocyte-specific genes, which lead to morphological changes and lipid accumulation within the cells. CA treatment successfully inhibited the expressions of these key adipogenic factors, PPARγ and C/EBPα, both in the *in vivo* model of HFD-induced obese mice and the *in vitro* adipocyte model, 3T3-L1 cells. Lipin is shown to be involved in various pathways regarding lipid metabolism in diverse cell types such as liver, adipose tissues, muscle, and neuronal cells (Phan and Reue, 2005; Finck et al., 2006; Reue and Zhang, 2008). Depletion of *LIPIN1* gene in preadipocytes delays fat cell differentiation, showing the importance of adipose-specific lipin-1 function in lipid homeostasis (Chen et al., 2008; Koh et al., 2008). Adipocyte fatty-acid-binding protien 2 (aP2) is a cytoplasmic chaperone which plays critical roles in several lipid signals (Tuncman et al., 2006). CA treatment decreased these lipogenic factors during the differentiation process in 3T3-L1 adipocytes.

Brown adipocytes are equipped with abundant mitochondria and uniquely express UCP1, which can dissipate the proton gradient of the inner mitochondrial membrane that is formed as a result of oxidative phosphorylation of nutrients. This process, also known as non-shivering thermogenesis, generates heat instead of ATP production (Cannon and Nedergaard, 2010). The transcriptional control network of brown adipogenesis has been analyzed and reviewed in detail elsewhere (Vernochet et al., 2010). In brief, the transcription factors PPARγ and C/EBPs including C/EBPα/β/δ have been established as essential parts of the differentiation of adipocytes. It is generally accepted that these transcription factors direct the differentiation of both brown and white adipocytes. The transcriptional events specifying brown adipogenesis are regulated by the nuclear co-activator PGC1α (Puigserver et al., 1998) and the transcription factor PRDM16, which physically interacts with C/EBPβ (Kajimura et al., 2009). Interestingly, recent evidence suggests that modulators of PPARγ-binding activity may specify brown adipogenic lineage transcriptional activity (Qiang et al., 2012). This modification is essential for recruitment of PRDM16 to PPARγ and initiation of the brown-fat-specific program (Qiang et al., 2012). CA was able to decrease lipid accumulation and increase *Ucp1*, *Pgc1a*, and *Sirt3* gene expressions in BAT of HFD-fed mice. Furthermore, in primary cultured brown adipocytes, CA treatment showed increased levels of mitochondria. Thermogenic factors including UCP1 and PGC1α were also up-regulated by CA treatment at both mRNA and protein levels as well, implying the thermogenic features of CA.

In differentiated fat cells, up-regulated SIRT1 promotes lipolysis resulting in loss of fat mass (Picard et al., 2004). Activation of the NAD^+^-dependent deacetylase SIRT1 by therapeutic small molecules, caloric restriction or exercise promotes mitochondrial biogenesis and activities (Milne et al., 2007), raising the possibility that SIRT1 can regulate BAT functions. SIRT1 and AMPK form a close relationship in the energy metabolic mechanism (Ruderman et al., 2010), and furthermore, AMPK is activated during cold exposure in C57BL/6 mice (Mulligan et al., 2007), implying the link between AMPK and BAT. Not only this, AMPK activation increases expressions of mitochondria-related genes through PGC1α expression (Jager et al., 2007). These previous reports support the relationship of AMPK and BAT activation. Our results showed that CA can suppress adipogenic factors in 3T3-L1 murine white adipocytes while it can increase thermogenic factors in primary cultured brown adipocytes. Though more investigation is required in order to reveal the exact mechanism, our results show that this multi-functioning effect of CA is possibly via the AMPK pathway as both features were nullified when AMPK activation was inhibited by CC treatment.

In the present study, we observed how the CA works on obese mice. The body weight, iWAT weight, eWAT weight, and liver weight was remarkably decreased in the CA administrated group. Furthermore, we discovered histological changes. The CA group was fed with HFD, same as the HFD-induced obese group, but by CA treatment, the morphology of WAT and BAT seemed like those of the NC group. The brown-fat-specific genes were also increased in BAT. The anti-adipogenic features of CA were confirmed using the zebrafish *in vivo* model. Then, we demonstrated that CA inhibited adipogenesis in 3T3-L1 cells. CA controlled adipogenesis factors, and interestingly, 3T3-L1 adipocytes co-treated with CA and Trog showed significantly nullified effect of Trog. This shows that CA is able to compete against PPARγ agonists. We also proved the effect of CA on thermogenic factors using primary cultured brown adipocytes. As expected, CA influenced primary cultured brown adipocytes by upregulating several thermogenic factors including UCP1, PGC1α, and PRDM16. Not only this, mitochondrial content was increased by CA treatment. From the increased levels of SIRT1 and SIRT3 in previous experiments, we hypothesized CA could affect AMPK activation. As expected, AMPK phosphorylation was increased by CA in 3T3-L1 cells. Furthermore, when we inhibited AMPK activation by CC pre-treatment, CA did not work properly in both 3T3-L1 adipocytes and primary cultured brown adipocytes. Through these results, we could discover that CA has anti-obesity effects *in vivo* and *in vitro*, and furthermore, were able to confirm the anti-obese effect was due to the activating effect of CA on the AMPK pathway.

## Author Contributions

HL, JP, H-LK, and M-YJ performed the *in vitro* experiments. HL, JP, JK, D-HY, and YJ performed the mouse model experiment. Y-IK and S-KC performed the zebrafish experiment. H-JK, KA, S-JK, and S-HH provided technical and material support. HL, JP, and H-LK collected the data. HL and JP wrote the manuscript. J-YU designed and supervised the study.

## Conflict of Interest Statement

The authors declare that the research was conducted in the absence of any commercial or financial relationships that could be construed as a potential conflict of interest.
